# Plant Growth-Promoting Rhizobacteria HN6 Induced the Change and Reorganization of *Fusarium* Microflora in the Rhizosphere of Banana Seedlings to Construct a Healthy Banana Microflora

**DOI:** 10.3389/fmicb.2021.685408

**Published:** 2021-07-20

**Authors:** Deyou Yang, Lanying Wang, Tianhao Wang, Yunfei Zhang, Shujing Zhang, Yanping Luo

**Affiliations:** Key Laboratory of Green Prevention and Control of Tropical Plant Diseases and Pests, Ministry of Education, School of Plant Protection, Hainan University, Haikou, China

**Keywords:** biocontrol factors, root colonization, rhizospheric microorganisms, microbial interaction, microbial diversity

## Abstract

*Streptomyces aureoverticillatus* HN6 was isolated in our previous study and effectively controlled banana *Fusarium* wilt. We explored the role of HN6 in constructing a healthy rhizosphere microflora of banana seedlings. The method of antibiotic resistance was used to determine the colonization ability of HN6. The effect of HN6 on the rhizosphere microbial communities was assessed using culture-dependent and high-throughput sequencing. The effect of HN6 on the infection process of the pathogen was evaluated using a pot experiment and confocal laser scanning microscopy. The results showed that HN6 could prevent pathogen infection; it increased the nutrient content and diversity of the bacterial community in the rhizosphere, promoted plant growth, and decreased the mycotoxin fusaric acid content and abundance of pathogens in the banana rhizosphere. Thus, HN6 decreased the relative abundance of *Fusarium* species, increased the diversity of fungi, and increased the relative abundance of bacteria in the rhizosphere. HN6 induced the change and reorganization of the microbial community dominated by *Fusarium* in the rhizosphere of banana seedlings, and it evolved into a community dominated that was not conducive to the occurrence of diseases, shaping the rhizosphere microflora and promoting the growth of banana.

## Introduction

The rhizosphere is a unique area that is limited to a few millimeters of soil that is in contact with the root system (Bais et al., 2006). The rhizospheric microbiome affects plant growth, development, and health (Berendsen et al., 2012; Wu et al., 2018). In the plant rhizosphere, plant roots absorb water and nutrients from the soil, and they affect the adjacent soil through root exudates (Zhang et al., 2017). Soil microorganisms play an important role in the formation and maintenance of healthy soils by regulating the biogeochemical cycle, organic matter reorganization, and mineralization (Beneduzi et al., 2012; Chen Y. et al., 2020).

Plant growth-promoting rhizobacteria (PGPR) provide an effective and environmentally sustainable method to protect crops against soil-borne pathogens (Walley, 1996; Cao et al., 2018). In the process of growth and reproduction, PGPR continuously produce bioactive substances, such as antibiotics, antibacterial proteins, and inhibitory volatile organic compounds (VOCs) (Viaene et al., 2016). Some rhizospheric microorganisms can also promote the release of soil nutrients through various metabolic activities and promote plant growth through the production of indole-3-acetic acid (IAA) and siderophore (Wang et al., 2017; Singh et al., 2018; Suleman et al., 2018).

Soil-borne pathogens substantially reduce yield in most agricultural systems, and they account for at least 50% of crop losses each year. Banana *Fusarium* wilt, also called banana Panama wilt, is a destructive soil-borne disease that affects bananas in tropical and subtropical areas worldwide (Dita et al., 2018). *Fusarium oxysporum* f. sp. *cubense* race 4 (FOC4) is one of the major pathogens that causes banana *Fusarium* wilt (Magdama et al., 2019; Maymon et al., 2020). It has been reported that the disease has seriously threatened the development of the global banana industry (Butler, 2013). *Bacillus*, *Streptomyces*, *Trichoderma*, and *Pseudomonas* spp. have been used against banana *Fusarium* wilt (Yuan et al., 2012; Sharma et al., 2018), and their use has been widely reported. Soil-borne pathogens substantially reduce yield in most agricultural systems, and they account for at least 50% of crop losses each year. The occurrence of soil-borne diseases is often associated with anomalies in the rhizosphere microbiota, including an increase in the number of harmful microbes and a decrease in the number of beneficial microbes. Biological control agents (BCAs) can inhibit the growth of soil-borne pathogens while restoring the rhizosphere microbial community. Actinomycetes are a class of Gram-positive bacteria that include many species with the potential for biocontrol of soil-borne plant diseases (Qin et al., 2011; Tan et al., 2016). *Streptomyces* are regarded as important biological control resources because of their bioactive secondary metabolites, and these antibacterial compounds play an important role in protecting plants against pathogens. *Streptomyces* occupies the main part in the study of bioactive compounds of Actinomycetes. In the study of bioactive compounds of *Streptomyces*, avermectin, tetracycline, streptomycin, nystatin, and erythromycin have been widely used in the fields of agriculture and medicine (Colombo et al., 2019). Mounting evidence suggests that the rhizosphere microbial community and its interactions with the plant root and rhizosphere environment determine plant health (Zhalnina et al., 2018; Chen D. et al., 2020). Therefore, the key to controlling the spread of soil-borne diseases is to understand the changes in plant rhizosphere microbial composition and microenvironment while maintaining the microbial diversity of the rhizosphere soil.

*Streptomyces aureoverticillatus* strain HN6 was originally isolated in Hainan, China. It is highly inhibitory to FOC4, which causes banana *Fusarium* wilt. The results of previous experiments showed that this strain exhibits broad spectrum antifungal activity and is strongly inhibitive to FOC4, the causal agent of banana *Fusarium* wilt in bio-assays (Wang et al., 2015). We sought to determine the link between the control of soil-borne diseases, promotion of plant growth, and shifts in the rhizosphere microbiota following application of the HN6 strain. A pot experiment was designed to apply the strain to the rhizosphere of banana seedlings to verify the control effect of strain HN6 on banana *Fusarium* wilt. The potential ability of the strain to change the soil nutrient elements of the root system was determined to clarify the role of the strain in the microbial interaction. The effects of the strain on the plant rhizosphere microenvironmental factors were determined by measuring the physical and chemical properties of the banana rhizosphere soil and the changes in mycotoxins. The bacterial 16S ribosomal RNA (rRNA) region and fungal internal transcribed spacer (ITS) region were used for high-throughput sequencing, and culturable microorganisms in the rhizosphere soil samples were isolated and identified. FOC4 in the rhizosphere soil samples was quantitatively detected by real-time PCR, and the effect of strain HN6 on the microbial community in the rhizosphere soil of susceptible banana seedlings was further evaluated. The mechanism for constructing healthy microflora in the banana seedling rhizosphere by strain HN6 was further revealed, which laid the foundation for the development and application of the strain.

## Materials and Methods

### Tested Microbial Strains

The biocontrol strain used in this experiment was *S. aureoverticillatus* HN6 (FJ911617). FOC4 (ATCC 76255, *Foc*⋅TR4-GFP) was used as the target pathogen of banana seedling susceptibility in the pot experiment, and the confocal laser scanning microscopy was used to observe the effect of infection with strain HN6 on FOC4 (Li et al., 2011; Dong et al., 2019). The target fungi consisted of FOC4 (ATCC 76255), *F. oxysporum* f. sp. *cucumebrium* (ATCC 42357), *Fusarium graminearum* Sehwabe (ATCC 60309), *Botryosphaeria dothidea* (ATCC 208828), *Colletotrichum musae* (Berk & Curt) Arx (ATCC 96167), *Botryodiplodia theobromae* (ATCC 96743), *Colletotrichum gloeosporioides* (Penzig) Penzig et Saccardo (ATCC MYA-456), *Rhizoctonia solani* (ATCC 10183), *Gibberella zeae* (schwein) Petch (ATCC 209501), and *Sclerotinia sclerotiorum* (ATCC 18687). The above target fungi were used to evaluate the antifungal activity of *Strain HN6* VOCs. The above strains were obtained from the College of Plant Protection, Hainan University.

### Banana Seedlings and Soil

The banana seedlings used in the pot experiment were Brazilian bananas (*Musa* sp. AAA Cavendish subgroup cv. Brazil) with three to four leaves, and they had a similar size and health; the seedlings were purchased from the tissue culture center of Danzhou city, Hainan province. In addition, the soil used in the pot experiment was collected from the healthy banana garden of the experimental base of the College of Plant Protection, Hainan University.

### Soil Colonization Capacity of Strain HN6

#### Resistance of HN6 to Antibiotics and Fungicides

Rifampicin, streptomycin, carbendazim, chlorothalonil, and gentamicin were added to Gause No. 1 agar medium at concentrations of 10, 30, 50, 70, and 100 μg ml^–1^. The Gause No. 1 agar medium included 20.0 g of soluble starch, 15 g of agar, 0.5 g of K_2_HPO_4_, 0.5 g of NaCl, 1 g of KNO_3_, 0.5 g of MgSO_4_ 7H_2_O, 0.01 g of FeSO_4_ 7H_2_O, 0.5 g of NaCl, and distilled water in 1,000 ml, pH 6.0 (Poomthongdee et al., 2015). Strain HN6 was spread onto the medium and cultured at 28°C for 5 days and included agar medium without any antibiotics or fungicides as a control. The initial concentration of spore suspension of HN6 used in this experiment was 1 × 10^5^ cfu ml^–1^ (Qi et al., 2019). Following the method described previously, the growth status of the strain was observed, and HN6 colonies were counted.

#### Determination of Soil Colonization Capacity

The colonization capacity of HN6 in soil was determined by antibiotic labeling method (Yun et al., 2018). Briefly, the soil was separated into sterile iron canisters and autoclaved at 121°C for 20 min. The sterile soil was placed in flowerpots (Φ = 18 cm). HN6 spore suspensions (100 ml, 1 × 10^5^ cfu ml^–1^) were added to the flowerpots by mixing with soil on the first day of the experiment. After 15 days, 1 g of soil was added to 9 ml of sterile water, and then, the solution was shaken for 20 min and left to stand for 30 min. The supernatant (1 ml) was diluted to 1 × 10^–4^, 100 μl of which was inoculated on Gause No. 1 agar plate containing rifampicin (12.5 μg ml^–1^) and carbendazim (10 μg ml^–1^), and the solution was incubated at 28°C for 7 days. Following the method described previously, the resulting bacterial colonies were enumerated. Unsterilized soil was used as a control, and all of the experiments were performed in triplicate (Xiong et al., 2020).

### Disease Suppression and Growth Promotion Ability of Strain HN6

#### Pot Culture Experiments

The pot experiment was conducted with an average temperature of 28°C and humidity of 70% in the experimental base of the College of Plant Protection, Hainan University from September to December 2019. Watering was done once every 2 days, and photoperiod condition was 12 h light/12 h dark. Healthy rhizosphere soil was collected from the banana garden and sifted through a 20-mesh sieve. Banana seedlings with three to four leaves were selected and rinsed with sterile water. These banana seedlings were planted in plastic pots containing 3,500 g of soil, and the root dipping method for the pathogen (Tinna et al., 2020). Banana seedlings were irrigated with spore suspension of HN6 once. Four treatment groups were set up, namely, N6 (inoculated with *Foc*⋅TR4-GFP + HN6, 1.0 × 10^7^ cfu g^–1^ soil), KY (inoculated with *Foc⋅*TR4-GFP, drenched with 0.1% carbendazim), KP (inoculated with *Foc*⋅TR4-GFP on sterile Gause No. 1 liquid medium), and KQ (inoculated with *Foc⋅*TR4-GFP, drenched with sterile water). YS was the initial soil, KY was the positive control, KP was the medium control, KQ was the sterile water treatment control, and N6 was the routine disease plus strain HN6 zone (protective treatment). Each treatment consisted of three replicates of 30 banana seedlings per replicate. Furthermore, separate lines were set up between the control and protective treatments. Soil adhering to the roots after shaking was defined as rhizosphere soil. After gentle shaking, the accumulator roots, the rhizosphere soil, free of roots, were carefully collected from each treatment. Each treatment was in three replicates, and 10 banana seedlings per replicate were used to obtain the pool of roots. Half of the fresh rhizosphere soil was used to determine biochemical properties and for the fusaric acid (FA) analysis, quantitative real-time PCR (qRT-PCR) of FOC4, and analysis of culturable microbiota. The other half was frozen with liquid nitrogen and then stored at −80°C to analyze microbial community structure (Baudoin et al., 2003).

Pot experiments were conducted in accordance with the methods described below. For *Foc⋅*TR4-GFP inoculation, an aseptic spore suspension was prepared and diluted to a suitable concentration. Briefly, the HN6 and pathogen were scrapped from the Gauze No. 1 and Potato Dextrose Agar (PDA) agar plates, respectively, and diluted to make the spore suspensions. Then, 200 ml of the spore suspension was inoculated into the soil so that the number of *Foc⋅*TR4-GFP spores in the soil was 1.0 × 10^5^ cfu g^–1^ soil before transplanting the banana seedlings. In the N6 treatment, strain HN6 was cultured in Gause No. 1 liquid medium for 2 days at 28°C. The concentration of the spore suspension was adjusted by a hemocytometer and diluted to a suitable concentration, and then, 200 ml of the strain HN6 spore suspension was inoculated into the soil so that the amount of strain HN6 in the soil was 1.0 × 10^7^ cfu g^–1^ soil after the banana seedlings were transplanted. The KY treatment group was treated with 200 ml of 0.1% carbendazim under the same treatment conditions. The KP and KQ treatment groups were treated with sterile Gause No. 1 liquid medium and 200 ml of sterile water under the same treatment conditions. The management measures for the experimental plot remained the same throughout the experimental period.

#### Disease Incidence, Disease Index, and Biocontrol Efficacy

In the pot experiment, the total leaf number and etiolated leaf number of banana seedlings in each treatment group were counted after 42 days, and the disease index of the banana seedlings and the incidence of banana plants in each treatment group were determined (Himaman et al., 2016). The grading standards of FOC4, the formula for disease incidence, disease index, and the biocontrol efficacy are shown as follows:

$$\mathrm{Disease}\mathrm{incidence}=\frac{\begin{array}{c} \mathrm{The}\mathrm{number}\mathrm{of}\mathrm{diseased}\mathrm{banana}\mathrm{seedlings}\\ \mathrm{in}\mathrm{each}\mathrm{treatment}\mathrm{group} \end{array}}{\begin{array}{c} \mathrm{The}\mathrm{total}\mathrm{number}\mathrm{of}\mathrm{banana}\mathrm{seedlings}\\ \mathrm{in}\mathrm{each}\mathrm{treatment}\mathrm{group} \end{array}}\times 100\%$$

$$\mathrm{Disease}\mathrm{index}=\frac{\begin{array}{c} \sum (\mathrm{Number}\mathrm{of}\mathrm{diseased}\mathrm{plants}\mathrm{of}\mathrm{each}\mathrm{grade}\\ \times \mathrm{value}\mathrm{of}\mathrm{relative}\mathrm{rade}) \end{array}}{\begin{array}{c} \mathrm{Total}\mathrm{number}\mathrm{inspected}\times 6 \end{array}}\times 100\%$$

$$\mathrm{Biocontrol}\mathrm{efficacy}=\frac{\begin{array}{c} \mathrm{Sterile}\mathrm{water}\mathrm{controlled}\mathrm{disease}\mathrm{index}-\\ \mathrm{treated}\mathrm{disease}\mathrm{index} \end{array}}{\begin{array}{c} \mathrm{Sterile}\mathrm{water}\mathrm{controlled}\mathrm{disease}\mathrm{index} \end{array}}\times 100\%$$

Grade 0, healthy plant; Grade 1, the leaves of the lower part of the plant were withered; Grade 3, 20–40% of the leaves were withered; Grade 4, 40–60% of the leaves were withered; Grade 5, 60–80% of the leaves were withered; and Grade 6, the entire plant was completely wilted and dead.

The inoculated banana plants were examined for infection and the effect of treatment with HN6 by *Foc*⋅TR4-GFP every day for the first 15 days. Five new plants of each treatment were selected for microscopic analysis each day, and five root samples per plant were harvested to investigate six different root areas that included the root hair, root cap, meristematic zone, elongation zone, and middle and basal part of the roots. For microscopic examination, the banana root tissue was prepared by first washing the roots in sterile distilled water to remove soil. The roots were then placed on a microscope slide, submerged in a water droplet, and covered with a glass coverslip.

#### Determination of Banana Physiological Indices

Various physiological indices including chlorophyll content, leaf area, plant height, stem diameter, and fresh and dry weights of the whole plant were measured at the 42nd day of the pot experiment (Chen et al., 2017). The chlorophyll content was measured by a Chlorophyll Assay Kit (Beijing Solarbio Science & Technology Co., Ltd., Beijing, China). Briefly, 0.1 g of fresh plant leaves was washed with distilled water. Then, the midrib was removed, and the leaves were cut and put in a homogenizer. One milliliter distilled water and 10 mg Reagent’ No. 1 were added under dark conditions, and the extract was transferred to a 10-ml test tube. Two hundred microliters of the upper extract was taken to determine the absorption value at 663 and 645 nm, which was recorded as A_663_ and A_645_, respectively. Following the method described previously, the formula used to calculate the chlorophyll content was as follows:

$$\mathrm{Chlorophyll}\mathrm{content}\left(\mathrm{mg}{\text{g}}^{-1}\right)=[0.01\times (33.7\times {\text{A}}_{645}+13.4\times {\text{A}}_{663})\times \text{F}]/\text{W},$$

where F is the dilution multiple and w is the sample weight (g).

### Effects of Strain HN6 on the Microenvironment of Rhizosphere Soil

#### Determination of Strain HN6 Enzymatic Activity

Using the bacterial cake inoculation method (Peng et al., 2020), strain HN6 cakes with a diameter of 5 mm by punch were inoculated on four different enzyme activity detection media, including lipase, protease, amylase, and cellulase. After 7 days, the presence and size of the halo and transparent rings were observed to detect lipase and protease activities. Lugol’s iodine solution was added to the amylase detection medium to observe the existence and size of the hydrolytic circle to detect amylase activity. Then, 2% Congo red solution was added to the cellulase detection medium, and the existence and size of the transparent circle around the colony were observed to detect the cellulase activity (Han et al., 2018).

#### Quantification of Indole-3-Acetic Acid Production

Tryptophan (0.05 g) and 1 ml of the spore suspension of HN6 (10^6^ cfu ml^–1^) were added to 100 ml of Gause No. 1 liquid medium, which was incubated at 28°C for 5 days with shaking at 160 rpm. Tryptophan was not added to the other two treatments. The culture was then centrifuged at 10,000 rpm for 10 min at 4°C. The supernatant (2 ml) was mixed with 2 ml of Salkowski reagent and incubated in the dark, and the development of color was measured (Glickmann and Dessaux, 1995). A standard curve was prepared by substituting the sample with IAA solution (5, 15, 20, 30, 40, 50, and 60 μg ml^–1^) using the Salkowski colorimetric method. The supernatant (2 ml) was added to 4 ml of Salkowski reagent and incubated in the dark at 25°C for 30 min. The samples with and without tryptophan were immediately measured at OD_530_, and the blank control was added after the addition of the colorimetric solution. Following the method described previously, the amount of IAA was calculated based on the standard curve.

#### Siderophore Production

The sterilized filter paper (Φ = 6 mm) were placed on chrome-azurol S (CAS) plates, seeded with the spore suspension of HN6 (10 μl, 10^6^ cfu ml^–1^), and incubated at 28°C for 5 days. The diameter of the orange zone around the filter paper was then measured (Machuca and Milagres, 2003).

#### Phosphate-Solubilizing Capacity

Using the molybdenum antimony anti-colorimetric method (Jing et al., 2016), potassium dihydrogen phosphate (KH_2_PO_4_) was used for the standard curve. The spore suspension of HN6 (0.5 ml, 10^6^ cfu ml^–1^) was added to 50 ml of montanine organophosphate liquid medium. The solution was incubated at 28°C for 5 days with shaking at 160 rpm, and then, it was centrifuged at 10,000 rpm for 10 min at 4°C. An equal amount of inactivated spore suspension was used as a control. The absorbance of 2 ml of supernatant was measured at OD_700_. Following the method described previously, phosphate solubilization was calculated based on the standard curve.

#### Quantification of Organic Acid Production

Starch was replaced with glucose in Gause No. 1 liquid medium, and the pH was adjusted to 7.0. The spore suspension of HN6 (1 ml, 10^6^ cfu ml^–1^) was inoculated into 100 ml of Gause No. 1 liquid medium, which was incubated at 28°C for 5 days with shaking at 160 rpm and then centrifuged at 8,000 rpm for 10 min at 4°C. The supernatant (10 ml) and methyl red reagent (three to five drops) were mixed, and the resulting color was observed. An equal amount of spore suspension, autoclaved at 121°C for 20 min, was used as control.

#### Measurement of Rate of Potassium Release

Spore suspension of HN6 (1 ml, 10^6^ cfu ml^–1^) was inoculated into 100 ml of Gause No. 1 liquid medium and then incubated at 28°C with shaking at 160 rpm for 3 days in a shake flask. The culture was mixed with Aleksandrov medium (1:0.04 v/v), where potassium feldspar was the only source of potassium, and then further cultured (Hu et al., 2006). An equal amount of inactivated seed culture was mixed with Aleksandrov medium (1:0.04 v/v) as the control. The entire culture was poured into an evaporating dish, heated, and concentrated to 15 ml. Hydrogen peroxide (4 ml) was added, the solution was stirred, and the mixture evaporated until it became a transparent liquid, which was then centrifuged at 8,000 rpm for 10 min at 4°C. The supernatant was then transferred into a volumetric flask and diluted with sterile water to a final volume of 50 ml (Meena et al., 2014). Following the method described previously, the potassium content was determined using a flame spectrophotometer. A standard curve was prepared using KCl solution at concentrations of 0, 2.5, 5.0, 10.0, 15.0, 20.0, and 40.0 μg ml^–1^. The following formula was used to calculate the rate of potassium release:

$$\mathrm{Potassium}\left(\%\right)=\frac{\text{a–b}}{\text{c}\times \text{d}\times 2\times 10^{4}}\times 100$$

where a refers to the amount of available potassium in the treatment (mg L^–1^), b is the amount of available potassium in the control (mg L^–1^), c is the weight of the potash feldspar (g), and d is the potassium content (%).

#### Volatile Organic Compounds-Mediated Antifungal Activity

The effect of strain HN6 VOCs on the growth of the target fungus was investigated using the bottom of one 90-mm diameter Petri dish to show physical separation between strain HN6 and the fungus. Fungal growth inhibition was calculated by measuring the radial growth of the fungal hyphae after 3 days of incubation. Percentage of inhibition was calculated as [(diameter of fungus in control − diameter of fungus exposed to VOCs) × 100/diameter of fungus in control] for each of the three replicates (Cordovez et al., 2015).

To trap the VOCs, the strain HN6 isolates were inoculated individually in 10-ml sterile glass vials containing 2.5 ml of Gause No. 1 liquid medium with three replicates each. Vials containing medium only served as controls. All of the vials were closed and incubated at 28°C. After 7 days, VOCs from the headspace of each vial were collected by solid phase microextraction (SPME) with a 65-mm polydimethylsiloxane-divinylbenzene fiber (Supelco, Bellefonte, PA, United States). Strain HN6 VOCs were analyzed by a gas chromatography-mass spectrometer (GC-MS) (Agilent GC7890A with a quadrupole MSD Agilent 5978C). VOCs were tentatively annotated by comparing their mass spectra with those of commercial (NIST08) and in-house mass spectral libraries (Santoro et al., 2016).

#### Effects of Strain HN6 on the Biochemical Properties of the Soil Samples

Using the same treatment method as that in the pot culture experiment, the rhizosphere soil of banana seedlings in different treatment groups was collected. The rhizosphere soil sample has been removed of impurities and stored at −80°C. The rhizosphere soil organic matter, rhizosphere soil total nitrogen, available phosphorus, and available potassium content were determined (Li et al., 2021).

#### Effects of Strain HN6 on the Fusaric Acid Content in Rhizosphere Soil

The FA content was determined based on a previously published method with minor modifications (Tan et al., 2011; Zhou J. et al., 2017). The concentrations of FA were 5, 10, 20, 30, 40, and 50 μg ml^–1^. The absorption spectrum and absorbance of FA at λ = 200–300 nm was determined using a UV-1100 spectrophotometer (MAPADA, Shanghai, China). The standard curve of FA was drawn with OD_272_ as ordinate and the concentration of FA as the horizontal coordinates. After weighing the rhizosphere soil samples, 5 ml of potassium dihydrogen phosphate solution (pH 2) was added into a conical bottle with 1 g of rhizosphere soil, followed by 30 ml of ethyl acetate, to extract for 30 min. After it was removed, the solute was filtered, and then, 5 ml of pH 2.5 potassium dihydrogen phosphate solution was added, followed by 30 ml of ethyl acetate for ultrasonic extraction for 30 min. After being removed, the solute was filtered again, and the crude toxin filtrate was collected. The OD of the sample was determined using a UV-1100 spectrophotometer (Wang et al., 2019, 2020). Following the method described previously, the content of FA in each rhizosphere soil sample was calculated using a standard curve.

#### Effects of Strain HN6 on the Number of FOC4 in Rhizosphere Soil

Quantitative real-time PCR was used to quantitatively measure the FOC4 biomass in the banana rhizosphere soil (Wang et al., 2018). The rhizosphere soil sample DNA was extracted using an E.Z.N.A.^®^ Soil DNA Kit (Omega Bio-tek, Norcross, GA, United States) per the manufacturer’s instructions. The specific primer set Foc-1/Foc-2 (5′-CAGGGGATGTATGAGGAGGCT/5′-GTGACAGC GTCGTCTAGTTCC) was used for PCR amplification. The description of PCR mixture and thermal cycling conditions used were the same as reported (Lin et al., 2009), and then, the rhizosphere soil sample DNA was diluted 10 times as a template and tested on the StepOne Plus type qRT-PCR instrument (ABI, Foster, CA, United States). The following reaction conditions were used to establish and optimize the qRT-PCR detection system and reaction procedure of FOC4. The amplification efficiency was 1.09, the slope of the curve was −3.104, and the linear equation was *y* = −3.546*x* + 44.241 (*R*^2^ = 0.999; *y* represents Ct, and *x* represents the logarithm of the DNA concentration). The standard curve was used for the detection of FOC4. According to the standard curve, the absolute quantitative detection copy number of the DNA concentration of FOC4 in the soil sample was calculated.

### Effects of Strain HN6 on the Microbial Community Structure in Rhizosphere Soil

#### Determination of the Microbial Community in Rhizosphere Soil

The total DNA of the soil microorganisms was extracted using an E.Z.N.A.^®^ Soil DNA Kit (Omega Bio-tek, Norcross, GA, United States) per the manufacturer’s instructions. The V3–V4 region of the 16S rRNA gene of bacteria was amplified using the universal primers 341F (5′-CCTACGGGNGGCWGCAG-3′)/80 5R (5′-GACTACHVGGGTATCTAATCC-3′), and the ITS1 region of fungi was amplified by ITS1F (5′-CTTGGTCATT TAGAGGAAGTAA-3′)/ITS1R (5′-GCTGCGTTCTTCATCGAT GC-3′). Finally, high-throughput sequencing (Illumina MiSeq) was used to analyze the structure of the microbial community. The method of analysis was mainly based on Shell scripts, combined with Python and R-related software packages, to realize the analysis of big data and generate actionable tables.

#### Determination of the Population of Culturable Rhizosphere Soil Microbes

The culture-dependent method was used to separate culturable fungi, bacteria, and actinomycetes from the rhizosphere soil samples that were collected by different selective media (Rossmann et al., 2012). Briefly, PDA, rose Bengal agar, and Martin agar medium were used for the fungi. The bacteria were cultured in nutrient agar medium, *Pseudomonas* selective, and Luria–Bertani agar medium. Actinomycetes were cultured in the medium of Gause No. 1 agar medium, starch casein agar, and humic acid–vitamin agar with 20 mg L^–1^ of nalidixic acid. The modified cetyl trimethylammonium bromide method and a bacterial genomic DNA extraction kit (Solarbio, Beijing, China) were used to extract total DNA to identify the fungi, bacteria, and actinomycetes (Huang et al., 2018). PCR amplification was carried out using the universal primers ITS1 and ITS4 (ITS1: 5′-TCCGTAGGTGAACCTGCGG-3′; ITS4: 5′-TCCTCCGCTTATTGATATGC-3′), as well as 27F and 1492R (27F: 5′-AGAGTTTGATCCTGGCTCAG-3′; 1492R: 5′-TACG GCTA CCTT GTTACGACTT-3′). The amplification profile included an initial denaturation at 95°C for 5 min, followed by 35 amplification cycles of 94°C for 1 min, 55°C for 1 min, and 72°C for 2 min. Finally, the PCR products were sequenced by using the primers 27F and 1492R, and the gene sequences were compared using BLAST in the NCBI database^1^. A phylogenetic tree was constructed using the neighbor joining method using MEGA version 7.0 (Luo et al., 2013).

### Data Analysis

Analysis of variance and multiple comparisons were carried out using SPSS 25 software by analysis of variance (ANOVA). The data were expressed as means ± standard deviation, and the significance was determined using Fisher’s protected least significant difference (LSD) test (*p* < 0.05).

## Results

### Colonization Ability of Strain HN6 in Soil

The resistance of HN6 to antibiotics and fungicides labeling method was used to determine the resistance of strain HN6 to three antibiotics and two fungicides, including rifampicin, gentamicin, streptomycin, carbendazim, and chlorothalonil. The results showed that the strain could grow under the condition of 10 μg ml^–1^ rifampicin, which was better than gentamicin and streptomycin, but it could not grow in the medium containing chlorothalonil (Table 1). Two kinds of antibiotics and two fungicides were selected for further test. The results showed that the strain grew the most under the combination of rifampicin (12.5 μg ml^–1^) and carbendazim (10 μg ml^–1^). The combined fungicidal concentration was used as the detection concentration of strain HN6, and a colonization test of the strain was carried out. After 15 days, the number of strain HN6 in sterilized soil was 9.4 × 10^4^ cfu g^–1^, and the number of strain HN6 in unsterilized soil was 2.3 × 10^4^ cfu g^–1^ (Table 2). The results showed that strain HN6 could colonize both sterilized and unsterilized soils, but it grew slower in the unsterilized soil. The reason may be that there were other microorganisms in unsterilized soil, and it takes a longer for strain HN6 to become dominant.

**TABLE 1:** Resistance of strain HN6 to antibiotics and fungicides.

Fungicides and antibiotics	Concentration (μg mL^–1^)	The number of HN6
Rifampicin	100	++
	70	+++
	50	+++
	30	+++
	10	+++
Gentamicin	100	–
	70	–
	50	–
	30	++
	10	++
Streptomycin	100	+
	70	++
	50	++
	30	++
	10	++
Carbendazim	100	+++
	70	+++
	50	+++
	30	+++
	10	+++
Chlorothalonil	100	–
	70	–
	50	–
	30	–
	10	–

*“–” means the HN6 unable to grow; “+” means the number of HN6 was 1–250 cfu g^–1^⋅soil; “++” means the number of HN6 was 250–500 cfu g^–1^⋅soil; and “+++” means the number of HN6 was more than 500 cfu g^–1^⋅soil.*

**TABLE 2:** Colonization ability of strain HN6 in soil (×10^4^ cfu g^–1^ soil^–1^).

	1st day	15th day	
Number of HN6 in soil		Sterilized soil	Unsterilized soil
Average	3.40 ± 0.82 Bb	9.40 ± 1.21 Aa	2.30 ± 0.75 Bb

*Data represent the means ± SD. Different lowercase letters in the same line show values that are significantly different at the *p* < 0.05 level, and different capital in the same line show values that are significantly different at the *p* < 0.01 by Duncan’s new multiple range test.*

### Disease Suppression and Growth Promotion Ability of Strain HN6

According to the statistics regarding the disease control effect of strain HN6 (Figure 2A), it was found that the disease degree and disease index of banana seedlings treated with strain HN6 were significantly lower than those of the control (*p* < 0.05), and the number of healthy banana seedlings was the largest in the treated trials (Table 3). Additionally, the lesion size in corms of banana seedlings treated with strain HN6 was less than that of the control (Supplementary Figure 1). The disease index of banana seedlings treated with only medium (KP) and sterile water (KQ) was 74.00 and 81.33. The disease index of banana seedlings treated with 0.1% carbendazim (KY) was 36.67, whereas that of banana seedlings treated with strain HN6 (N6) was 11.33, which was lower than that of treatments KP and KQ. The percentage of biocontrol efficacy was 86.09.

**TABLE 3:** Disease incidence, disease index, and biocontrol efficacy.

Treatment	Disease incidence	Disease index	Biocontrol efficacy (%)
N6	13.33 ± 3.34c	11.33 ± 1.53d	86.09
KY	84.44 ± 5.59b	36.67 ± 2.00c	54.92
KP	96.67 ± 3.34a	74.00 ± 2.00b	–
KQ	97.78 ± 1.92a	81.33 ± 1.53a	–

*Data represent the means ± SD. Different lowercase letters in the same column show values that are significantly different at the *p* < 0.05 level by Duncan’s new multiple range test.*

**FIGURE 1:**
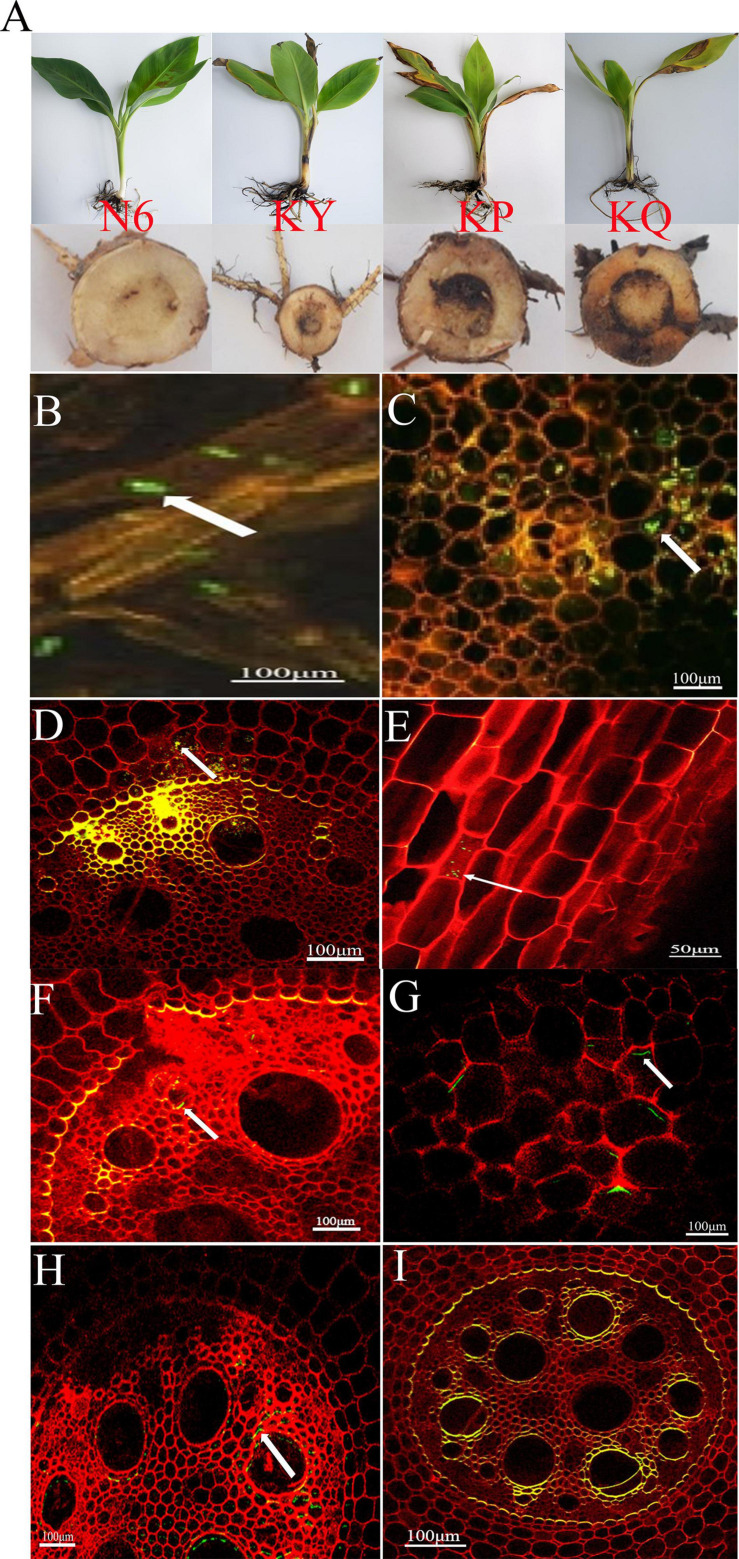
The effects of strain HN6 on the infection process of *Foc* TR4-GFP. N6 (*Foc*⋅TR4-GFP + HN6, 1.0 × 10^7^ cfu g^–1^ soil), KY (*Foc*⋅TR4-GFP + 0.1% carbendazim), KP (*Foc*⋅TR4-GFP + Gause No. 1 liquid medium), and KQ (*Foc* TR4-GFP + sterile water). YS referred to rhizosphere soil of uninoculated plants. **(A)** Susceptible banana seedlings. **(B)** At the first day postinfection, hyphae and spores adhered to root hair and root epidermis of the KQ bananas. **(C)** At the seventh day postinfection, *Foc* TR4-GFP spores were observed on the root hairs and also in the parenchymal cells of the KQ banana roots. **(D)** At the 11th day postinfection, the hyphae extended upward along root vascular bundles to corms of the KQ bananas. **(E)** Direct penetration of epidermal cells of the KQ bananas roots at the 15th day; **(F,G)** N6 and KY groups: at the 15th day, no hyphae were found in the corm central cylinder tissues of the bananas; KP and KQ groups: **(H,I)** at the 15th day, hyphae and spores adhered to the corm central cylinder tissues of the bananas.

The effects of strain HN6 on the infection process of *Foc* TR4-GFP were observed using confocal laser scanning microscopy. The results showed that at the first day postinfection, hyphae and spores adhered to root hair and root epidermis of the KQ treatment bananas (Figure 1B), and at the seventh day postinfection, *Foc* TR4-GFP spores were observed on the root hairs and also in the parenchymal cells of banana roots (Figure 1C). At the 11th day postinfection, the hyphae extended upward along root vascular bundles to corms of KQ treatment bananas (Figure 1D). At the 15th day postinfection, the spore invasion of *Foc* TR4-GFP was observed in the cells of the longitudinal section of the root in KQ treatment bananas (Figure 1E). At the 15th day postinfection, no hypha was found in corm central cylinder tissues of N6 (Figure 1F) and KY (Figure 1G), but a lot of hyphae grew in that of KP (Figure 1H) and KQ (Figure 1I). These results suggested that with the addition of strain HN6, *Foc* TR4-GFP was inhibited, and the infection ability was affected.

**FIGURE 2:**
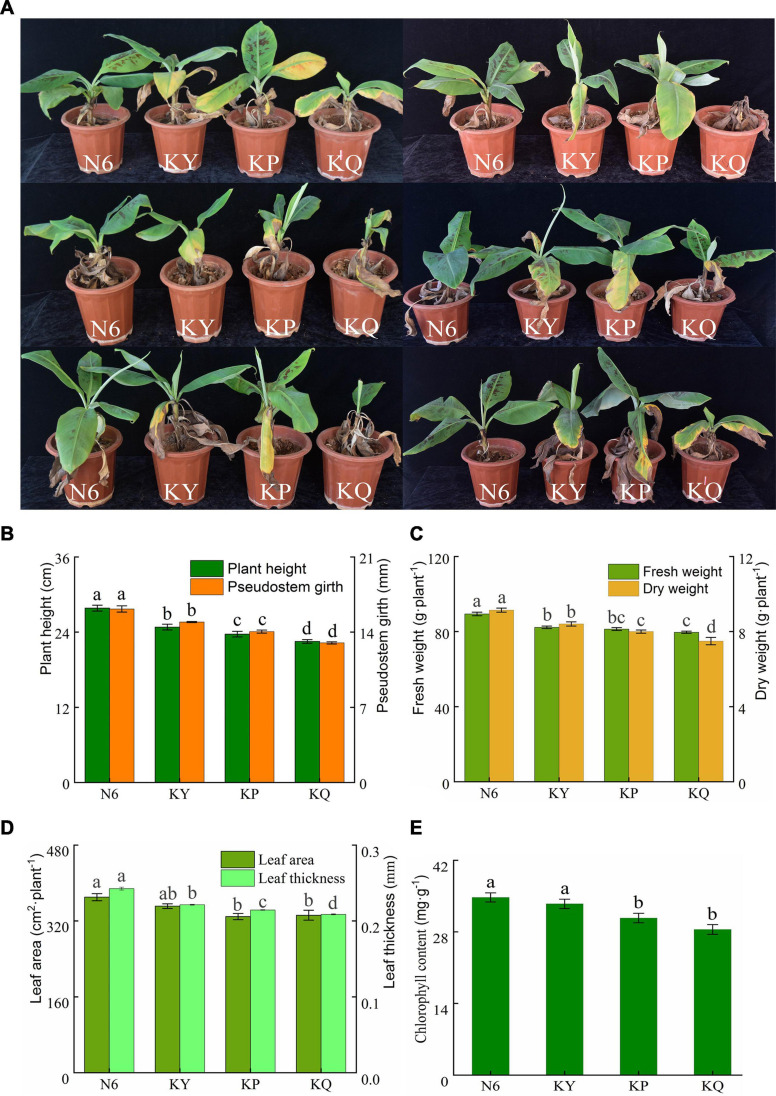
The biocontrol effects of strain HN6 against FOC4. N6 (*Foc*⋅TR4-GFP + HN6, 1.0 × 10^7^ cfu g^–1^ soil), KY (*Foc*⋅TR4-GFP + 0.1% carbendazim), KP (*Foc*⋅TR4-GFP + Gause No. 1 liquid medium), and KQ (*Foc* TR4-GFP + sterile water). YS referred to rhizosphere soil of uninoculated plants. **(A)** The incidence of banana seedlings in different treatment groups. **(B)** Plant height and pseudostem girth. **(C)** Fresh and dry weights. **(D)** Leaf area and leaf thickness. **(E)** Chlorophyll content. Error bars represent standard deviations, and means with different letters are significantly different from each other (*p* < 0.05) according to the least significant difference (LSD) test (*n* = 3).

The plant height, stem diameter, leaf area, and leaf thickness of the banana seedlings in each treatment group were measured. The results showed that strain HN6 could promote the growth of banana seedlings. The strain increased plant height, stem diameter (Figure 2B), fresh and dry weights (Figure 2C), leaf area and thickness (Figure 2D), and chlorophyll content (Figure 2E).

### Effects of Strain HN6 on the Microenvironment of Rhizosphere Soil

#### The Potential of Strain HN6 to Change Root Nutrients

**FIGURE 3:**
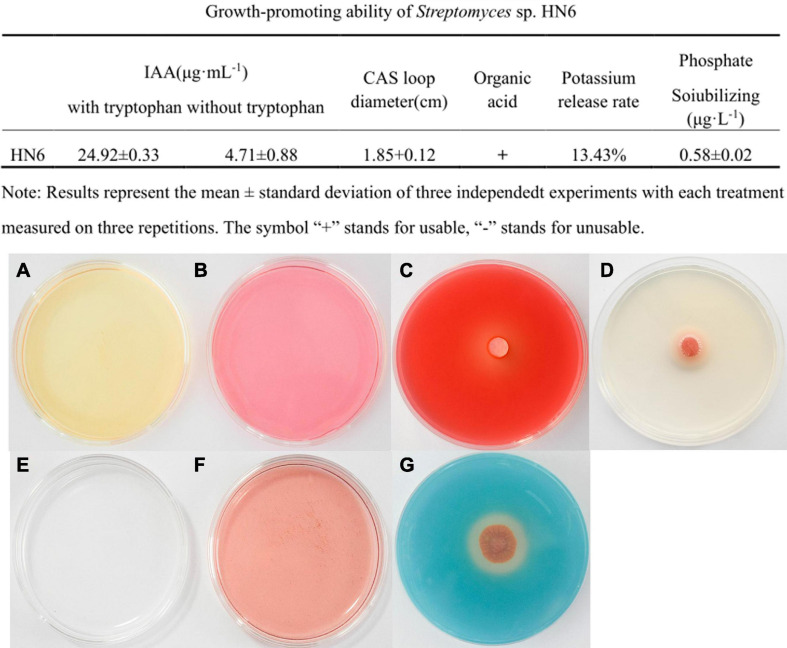
Plant growth promotion abilities of strain HN6. **(A,B)** IAA production, the strain could produce IAA in Gause No. 1 liquid medium in the absence or added of tryptophan. **(C)** Cellulose activity. An obvious halo and transparent circles were observed around the HN6 on the cellulase detection plate. **(D)** Lipase activity. An obvious halo and transparent circles were observed around the HN6 on the lipase detection plate. **(E,F)** Produces organic acid. **(G)** Siderophore production. On the brilliant blue chrome-azurol S plates, strain HN6 colonizes the diameter of the orange zone.

At 530 nm, the equation for the standard curve of IAA was *y* = 0.0276*x* − 0.0687 (*R*^2^ = 0.9971). According to the standard curve, the strain could produce IAA in Gause No. 1 liquid medium in the absence of tryptophan, which was yellow in color (Figure 3A). The IAA yield from the strain was 4.71 ± 0.08 μg ml^–1^. When tryptophan was added to the Gause No.1 agar plate, the strain could produce IAA, which was pink in color (Figure 3B). The IAA yield from the strain was 24.92 ± 0.33 μg ml^–1^, which was 5.29 times more than that in the absence of tryptophan, when incubated with yellow Salkowski reagent under dark conditions. These results indicated that strain HN6 effectively utilized exogenous tryptophan to produce IAA.

An obvious halo and transparent circles were observed around the bacterial cake on the cellulase and lipase detection plate. The diameter of the cellulase transparent ring was 1.91 ± 0.05 cm (Figure 3C), while the diameter of the lipase halo was 1.89 ± 0.16 cm (Figure 3D). In the organic acid test, the control medium did not turn red (Figure 3E) without inoculation with strain HN6, and the test solution turned red (Figure 3F) after inoculation with strain HN6, indicating that acidic substances were produced. Organic acid production is the principal mechanism adopted by phosphate-solubilizing bacteria for inorganic phosphate solubilization. On the brilliant blue CAS plates, strain HN6 colonizes the diameter of the orange zone averaged 1.85 cm (Figure 3G). According to the KCl standard curve, *y* = 0.0536*x* − 0.4456 (*R*^2^ = 0.996); the potassium release rate of the strain was 13.43%. This indicated that this strain is capable of dissolving potassium salts.

#### Effect of Strain HN6 VOCs on Fungal Growth

For testing the antifungal activity of VOCs produced by strain HN6, the hyphal growth of target fungi was measured during exposure to VOCs. The VOCs produced by HN6 showed antifungal activity on 10 different fungal species (Supplementary Figure 2). The best inhibition rate was for FOC4 (63.11%), followed by *B. dothidea* (35.21%) and *R. solani* (35.19%). The inhibition rate of strain HN6 VOCs to other pathogens were <30% (Table 4). A comparison of the VOC profiles of strain HN6 with the control pinpointed 33 VOCs potentially involved in its antifungal activity against FOC4 (Table 5). The VOCs detected from the strain were researched, and five common VOCs were selected {acridine, 9-methyl-; hexadecane; 3,3-bis[p-(dimethylamino) phenyl]-6-(dimethylamino) phthalide; thiophene,2-ethyl-; and hentriacontane}. Subsequently, different concentrations of these five VOCs were used to test their inhibitory effect on hyphal growth of FOC4. The VOC acridine, 9-methyl-, inhibited radial hyphal growth of FOC4. The EC_50_ was 109.824 μg ml^–1^.

**TABLE 4:** Antifungal activity of strain HN6 volatile organic compounds.

Pathogenic fungal	Inhibition rate (%)
*Fusarium oxysporum* f. sp. *cubense* Race 4	63.11 ± 1.91a
*Botryosphaeria dothidea*	35.21 ± 9.32b
*Rhizoctonia solani*	35.19 ± 1.58b
*Fusarium oxysporum* f. sp. *cucumebrium*	24.34 ± 7.58c
*Gibberella zeae* (schwein) Petch	23.82 ± 8.48c
*Botryosphaeria dothidea*	23.22 ± 7.97c
*Colletotrichum gloeosporioides*	20.36 ± 8.52c
*Sclerotinia sclerotiorum*	20.30 ± 0.18c
*Colletotrichum musae* (Berk & Curt) Arx	8.72 ± 1.51d
*Fusarium graminearum* Sehwabe	7.97 ± 3.27d

*Data represent the means ± SD. Different lowercase letters in the same column show values that are significantly different at the *p* < 0.05 level by Duncan’s new multiple range test.*

**TABLE 5:** Possible VOCs from HN6 using the area normalization method by SPME-GC-MS analysis.

No.	Possible compounds	RT	RA (%)
1	2-pinene, (1R,5R) – (+)-(8CI)	4.942	2.78
2	Acridine, 9-methyl-	5.534	4.18
3	Heptane, 3,5-dimethyl-	6.273	2.57
4	2-methyl-2-bornene	6.918	29.99
5	Hexadecane, 2,6,10,14-tetramethyl-	7.767	0.41
6	Octane, 2,3,6,7-tetramethyl-	7.872	1.99
7	Decane, 4-ethyl-	7.935	7.18
8	Decane, 2,3,7-trimethyl-	8.071	2.61
9	Octane, 6-ethyl-2-methyl-	8.837	0.30
10	Hexadecane	8.963	0.53
11	Undecane, 4-methyl-	9.01	1.63
12	9H-9-silafluorene, 9,9-dimethyl-4-(trimethylsilyl)-(9CI)	9.214	0.57
13	Cyclohexane, 1,2,4- trimethyl-, (1R,2R,4R)-rel-	9.995	0.50
14	Nonadecane, 9-methyl-	10.645	0.22
15	Cyclohexene, 1,6,6-trimethyl-	10.729	7.09
16	2-norbornanol, 1,2,7,7-tetramethyl- (6CI,7CI)	10.976	5.45
17	Cyclopentane, 1-hexyl-3-methyl-	11.154	1.27
18	Cyclohexane, 1,4-bis(methylene)-	12.034	1.09
19	Rosifoliol	12.218	0.71
20	1-tridecyn-4-ol	12.401	1.29
21	Eicosane, 1-iodo-	13.193	1.31
22	Hentriacontane	13.513	0.46
23	Octane, 2,4,6-trimethyl-	14.22	0.27
24	Bicyclo (3.1.1) heptan-2-ol, 4,6,6-trimethyl-	14.467	0.34
25	Nonadecane, 3-ethyl-3-methyl-	14.823	0.44
26	2-(benzyloxymethyl)-2-(but-3-enyl) cyclohexanone	14.986	0.40
27	Acetic acid, 2,2,2- trifluoro-, pentadecyl ester	15.274	0.32
28	Cyclotetradecane	15.657	0.15
29	3,3-bis[p-(dimethylamino) phenyl]-6-(dimethylamino)phthalide	15.714	0.12
30	2,3-dihydro-4-(1-methylethyl)furan	16.081	0.13
31	Thiophene, 2-ethyl-	17.502	1.20
32	Cyclohexane, 1-methyl-2-propyl	17.811	2.43
33	3,6-Dithiocyanatocarbazole	22.193	0.54

*RT, retention time; RA, relative peak area (%).*

#### Effect of Strain HN6 on the Nutrient Content of Rhizosphere Soil

**FIGURE 4:**
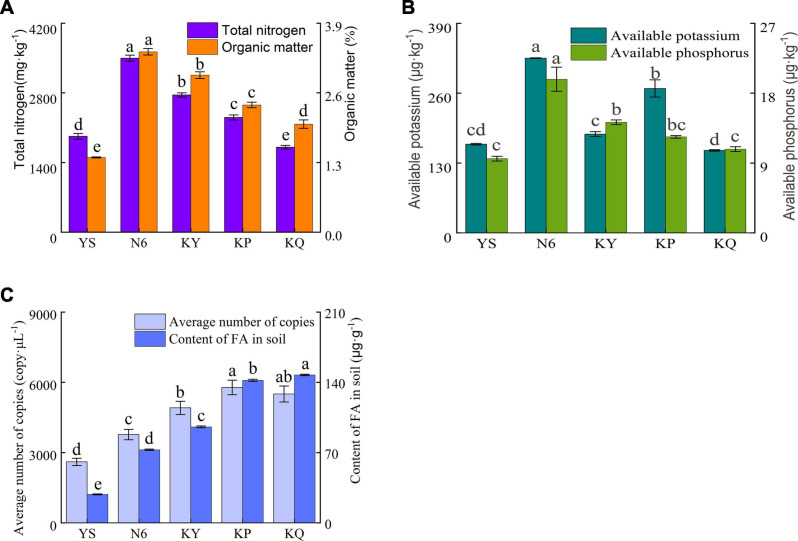
Effects of strain HN6 on the nutrient, FOC4, and fusaric acid contents of rhizosphere soil. N6 (*Foc*⋅TR4-GFP + HN6, 1.0 × 10^7^ cfu g^–1^ soil), KY (*Foc*⋅TR4-GFP + 0.1% carbendazim), KP (*Foc*⋅TR4-GFP + Gause No. 1 liquid medium), and KQ (*Foc* TR4-GFP + sterile water). YS referred to rhizosphere soil of uninoculated plants. **(A)** Total nitrogen and organic matter content of rhizosphere soil sample. **(B)** Available phosphorus and available potassium content of rhizosphere soil sample. **(C)** FOC4 and FA content of rhizosphere soil sample. Error bars represent standard deviations, and means with different letters are significantly different from each other (*p* < 0.05) according to the LSD test (*n* = 3).

According to the total nitrogen and organic matter (Figure 4A), available phosphorus, and available potassium (Figure 4B) results in the rhizosphere soil, compared with the rhizosphere soil of uninoculated plants (YS) and other treatments, the strain HN6 treatment increased the content of soil nitrogen and organic matter, available phosphorus, and available potassium, indicating that the strain can effectively increase the content of nitrogen in rhizosphere soil, improve soil nutrition, and promote plant growth. Compared with the control, the contents of total nitrogen, organic matter, available phosphorus, and available potassium were significantly increased in the *Streptomyces* sp. treatment group.

#### Effects of Strain HN6 on FOC4 Genomic DNA and Fusaric Acid Content in the Rhizosphere Soil

The average copy number of FOC4 DNA in the N6 treatment group was significantly lower (*p* < 0.05) than that in the control groups (Figure 4C), as was the content of the mycotoxin FA. In terms of FOC4 genomic DNA content, N6, KY, KP, KQ, and YS were 3,771.03, 4,909.73, 5,783.34, 5,502.50, 2,604.65 copy μl^–1^. KY, KP, and KQ control were 1.30, 1.53, and 1.45 times more of the N6 treatment. In terms of FA content, N6, KY, KP, KQ, and YS were 72.87, 95.66, 147.41, 142.06, and 28.36 μg g^–1^. KY, KP, and KQ control were 1.31, 2.02, and 1.95 times more of the N6 treatment.

### Effects of Strain HN6 on Microbial Composition in Rhizosphere Soil

#### Alpha Diversity Analysis

The indices of community richness are the Chao index and ACE index. The indices of community diversity are the Shannon index, Simpson index, and Coverage index. Among them, the higher the Chao and ACE index, the richer the community richness in the sample. The higher the Shannon index, the higher the community diversity in the sample. The higher the Simpson index, the lower the community diversity in the sample (Meng et al., 2020). The Chao coefficient and ACE index of the samples treated with strain HN6 (N6) were lower than those of the medium samples (KP) and sterile water control samples (KQ) (Table 6). The results showed that the community richness of fungi in the samples treated with strain HN6 decreased. In terms of community diversity, the Shannon index of the strain HN6-treated samples (N6) was significantly higher than that of the medium samples (KP) and sterile water control samples (KQ), Simpson index was significantly lower in HN6-treated samples, and the community diversity was the highest in the HN6 treatment group. The abundance and diversity of a microbial community is reflected by sample diversity analysis. The results showed that after strain HN6 treatment, the rhizosphere soil diversity was restored, and rhizosphere soil microecology was maintained.

**TABLE 6:** Statistical table of alpha diversity index.

Sample ID	Shannon index	ACE index	Chao index	Simpson	Coverage
YS	4.47 ± 0.04a	1,167.27 ± 86.32d	1,116.31 ± 101.62d	0.06 ± 0.01d	1.00 ± 0.00a
N6	3.85 ± 0.40b	1,378.25 ± 56.18*cd*	1,316.99 ± 43.08*cd*	0.12 ± 0.02c	1.00 ± 0.01*ab*
KY	3.47 ± 0.34*bc*	1,590.09 ± 82.88c	1,536.03 ± 74.50c	0.15 ± 0.02c	0.99 ± 0.01*ab*
KP	3.21 ± 0.18c	4,166.06 ± 160.67b	3,036.45 ± 141.61b	0.35 ± 0.02b	0.99 ± 0.01*ab*
KQ	2.34 ± 0.17d	5,461.22 ± 462.99a	3,936.10 ± 346.59a	0.45 ± 0.03a	0.99 ± 0.00b

*N6 (*Foc*⋅TR4-GFP+ HN6, 1.0 × 10^7^ cfu g^–1^ soil), KY (*Foc*⋅TR4-GFP + 0.1% carbendazim), KP (*Foc*⋅TR4-GFP+ Gause No. 1 liquid medium), and KQ (*Foc* TR4-GFP+ sterile water). YS referred to rhizosphere soil of uninoculated plants. Data represent the means ± SD. Different lowercase letters in the same column show values that are significantly different at the *p* < 0.05 level by Duncan’s new multiple range test.*

#### Effects on the Rhizosphere Soil Fungal Community Structure

**FIGURE 5:**
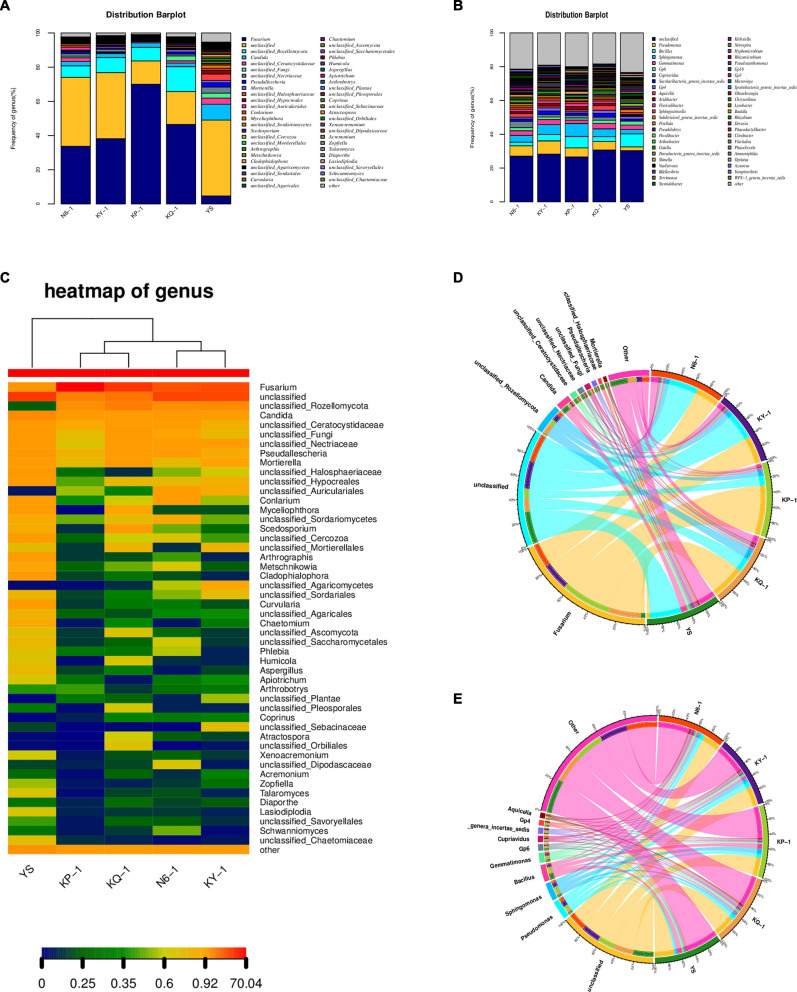
Effect of strain HN6 on the composition of rhizosphere soil microbial community. N6 (*Foc*⋅TR4-GFP + HN6, 1.0 × 10^7^ cfu g^–1^ soil), KY (*Foc*⋅TR4-GFP + 0.1% carbendazim), KP (*Foc*⋅TR4-GFP + Gause No. 1 liquid medium), and KQ (*Foc* TR4-GFP + sterile water). YS referred to rhizosphere soil of uninoculated plants. **(A)** Fungi community structure. **(B)** Bacterial community structure. **(C)** Heatmap of fungal genera. **(D)** Sample–species relationship diagram of fungi at genus level. **(E)** Sample–species relationship diagram of bacteria at genus level.

The operational taxonomic unit (OTU) sequence was compared with the corresponding database (RDP database^2^) and filtered to select the most suitable alignment result for the OTU sequence the results by comparison. The sequences with a similarity >90% and coverage >90% by default were used for subsequent classification, and the sequences that did not meet the conditions were classified as unclassified. According to the results of taxonomic analysis, the community composition of each sample at the genus level was counted. A community composition distribution map was obtained (Figure 5A), together with a heatmap of the genus (Figure 5C) and a sample–species relationship diagram at genus level. It can be found in the sample–species relationship diagram that the abundance of *Fusarium* was different in each sample, and the proportion of *Fusarium* in N6, KY, KP, KQ, and YS was roughly 16, 20, 36, 24, and 4% (Figure 5D). By analyzing the main dominant fungal species, it was found that the six genera with the highest abundance in the rhizosphere soil of uninoculated plants (YS) were unclassified *Candida*, *Fusarium*, unclassified*_Halosphaeriaceae*, unclassified*_Ceratocystidaceae*, and unclassified *Nectriaceae*. For the N6 treatment group, the six genera with the highest abundance were *Fusarium*, unclassified, *unclassified_Rozellomycota*, *Candida*, *unclassified_Ceratocystidaceae*, and *unclassified_Fungi*. According to the analysis results, the relative abundance of *Fusarium* in the YS was relatively low. When strain HN6 colonized the rhizosphere soil, compared with the control, the abundance of *Fusarium* is still the lowest. The results showed that strain HN6 had a strong inhibitory effect on the growth of *Fusarium* spp. in the rhizosphere of susceptible banana plants, which were consistent with the results of the alpha diversity analysis.

#### Effect on Rhizosphere Soil Bacterial Community Structure

The bacterial community structure distribution map was obtained by counting the bacterial community composition of each sample and the sample–species relationship diagram at the genus level. By analyzing the main dominant bacteria, it was found that the six genera with the highest abundance in the rhizosphere soil samples were unclassified, *Bacillus*, *Pseudomonas*, *Sphingomonas*, *Gemmatimonas*, and *Gp6*. According to the analysis results, the relative abundance of *Pseudomonas* in the rhizosphere soil of uninoculated plants was relatively low. When strain HN6 colonized the rhizosphere soil, the abundance of *Pseudomonas* increased (Figure 5B). It can be found in the sample–species relationship diagram that the abundance of *Pseudomonas* and *Bacillus* were different in each sample; the proportion of *Pseudomonas* in N6, KY, KP, KQ, and YS was roughly 6, 7, 5, 3, and 2%; and the proportion of *Bacillus* was the largest in N6 (Figure 5E).

#### Effects of Strain HN6 on Culturable Microorganisms in Rhizosphere Soil

**FIGURE 6:**
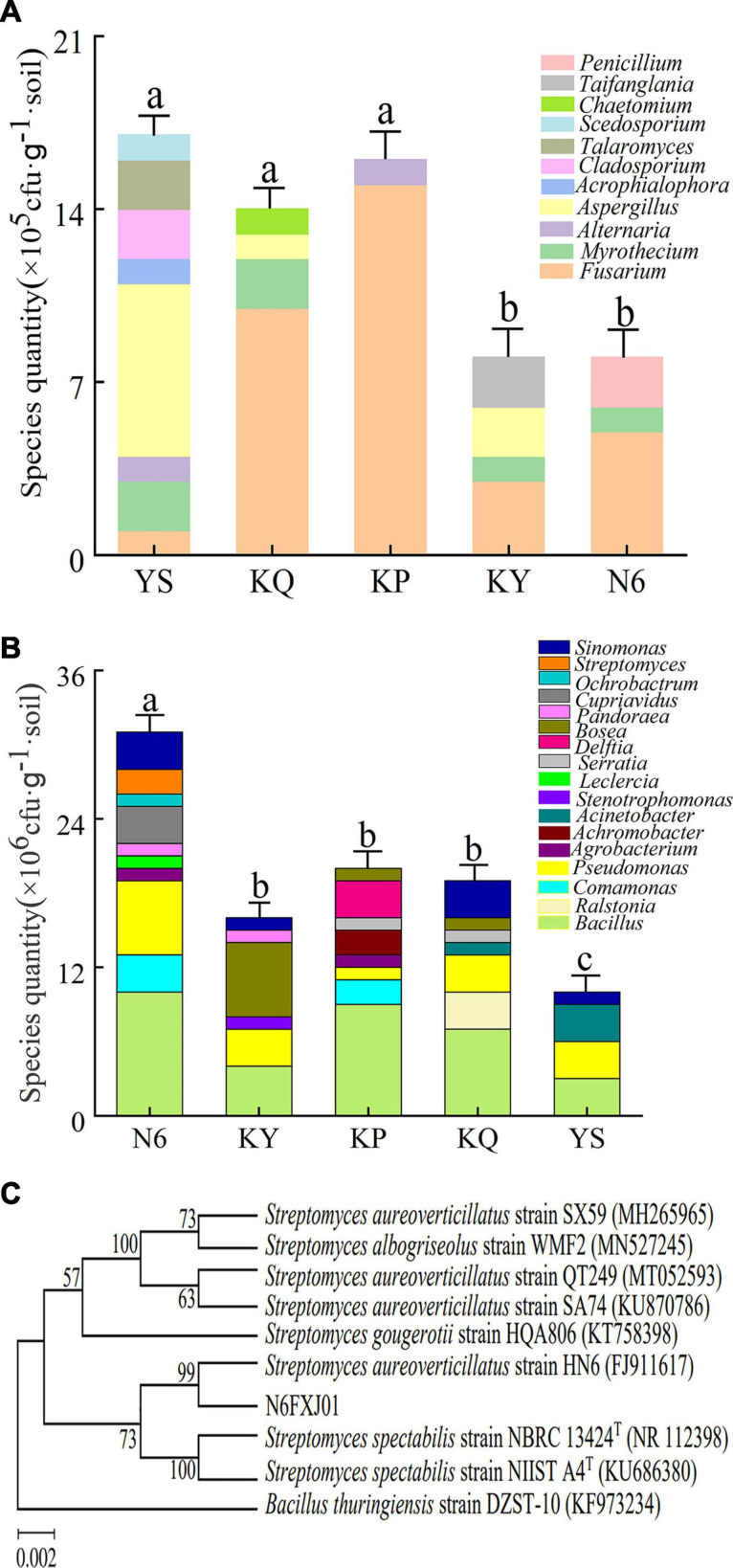
Structural composition of culturable microorganisms in rhizosphere soil. N6 (*Foc*⋅TR4-GFP + HN6, 1.0 × 10^7^ cfu g^–1^ soil), KY (*Foc*⋅TR4-GFP + 0.1% carbendazim), KP (*Foc*⋅TR4-GFP + Gause No. 1 liquid medium), and KQ (*Foc* TR4-GFP + sterile water). YS referred to rhizosphere soil of uninoculated plants. **(A)** Structural composition of culturable fungi. **(B)** Structural composition of culturable bacteria. **(C)** Phylogenetic analysis of strain N6FXJ01.

According to the results of the statistical, the number of culturable fungi in the N6 treatment group was less than that in the YS samples (Supplementary Table 1), and the number of culturable bacteria in the N6 treatment group was more than that in the control (Supplementary Table 2). The results of the sequence alignment analysis showed that after banana seedlings were infected by FOC4, *Fusarium* spp. became the dominant fungus in the rhizosphere, and the number of *Fusarium* in each treatment group was much higher than that in the original soil samples. Due to the antagonistic effect of strain HN6, the number of *Fusarium* on culturable microorganisms in rhizosphere soil increased to a lesser extent in the N6 treatment group (Figure 6A), which was consistent with the results of high-throughput sequencing. Through the analysis of the species and abundance of culturable bacteria in different genera, it was observed that the abundance of *Bacillus* and *Pseudomonas* in the N6 treatment group was higher than that in the other treatment groups (Figure 6B), indicating that strain HN6 could attract *Bacillus* and *Pseudomonas* to colonize the banana plant rhizosphere. In the culturable bacteria, a *Streptomyces* sp. N6FXJ01strain isolated from the N6 group was compared and analyzed by phylogenetic analysis. It was phylogenetically closely related to *S. aureoverticillatus* HN6 (FJ911617), which share 99.8% 16S rRNA sequence similarity (Figure 6C).

## Discussion

Although decades of research have been carried out on banana *Fusarium* wilt, there are few effective control methods for this disease, and the breeding of disease-resistant varieties is the most effective management strategy (Tao et al., 2020). However, these kinds of biological resources are usually scarce, unproductive, and commercially unacceptable (Dita et al., 2010). It is difficult to breed disease-resistant varieties using genetic transformation technology through conventional breeding. Biological control with antagonistic bacteria can provide potential prospects for sustainable plant protection (Shen et al., 2015). Shen et al. (2015) found that the application of *Bacillus amyloliquefaciens* for 2 years in an area where banana *Fusarium* wilt occurred could regulate the banana rhizosphere microflora and effectively control *Fusarium* wilt caused by *F. oxysporum* and increase the yield. *B. amyloliquefaciens* SQR9, which was isolated from the cucumber rhizosphere, suppressed the growth of *F. oxysporum* in the cucumber rhizosphere and protected the host plant from pathogen invasion through efficient root colonization (Xu et al., 2014). Seventeen strains of *Streptomyces* were isolated and promoted plant growth by producing an iron carrier, IAA, and dissolved phosphate (Baoune et al., 2018). Some researchers have also found that microbial VOCs display versatile functions: they inhibit bacterial and fungal growth, promote or inhibit plant growth, trigger plant resistance, and attract other micro- and macroorganisms (Hagai et al., 2014; Schmidt et al., 2015). VOCs indirectly improve plant growth by alleviating biotic and abiotic stress. Some VOCs, such as dimethyl disulfide (DMDS) and 2-methyl-pentanoate, are highly toxic to plant pathogens (Groenhagen et al., 2013; Cordovez et al., 2015; Raza et al., 2016; Ossowicki et al., 2017), and some, such as acetoin, 2,3-butanediol, and tridecane, induce plant systemic resistance (ISR) against these pathogens (Lee et al., 2012). However, ISR appears to be the main mechanism of disease suppression *via* VOCs under natural conditions (Sharifi and Ryu, 2016). Some VOCs can also induce systemic tolerance to rhizosphere soil salinization and drought stress, which pose major threats to crop production. Treatment with rhizobacteria can help alleviate these problems by improving root system architecture for more efficient water uptake. Rhizobacteria confer systemic tolerance to abiotic stress by modulating proline, antioxidant, and hormone production and reducing Na^+^ accumulation in plants (Liu and Zhang, 2015). Therefore, *Streptomyces* are regarded as important biological control resources because of their bioactive secondary metabolites, and these antibacterial compounds play an important role in protecting plants against pathogens.

*Streptomycetes* are widely distributed in the soil, where they are strong competitors and antagonists (Bubici et al., 2019). The use of *Streptomycetes* as BCAs is largely documented in the literature. Qin et al. (2010) selected 8 out of 139 isolates using *in vitro* assays against several *F. oxysporum formae speciales* and demonstrated that the application of their fermentation broth provided a *Fusarium* wilt of banana control ranging from 78 to 87% in pot experiments. In particular, using 1.85 × 10^6^ conidia ml^–1^ of FOC4, plants treated with the best *Streptomycetes* strain, ZJ-E1-2, showed *Fusarium* wilt of banana incidence of 12%, while it was 76% on untreated trees. Zhou D. et al. (2017) observed a *Fusarium* wilt of banana reduction of 73% after treatment with *Streptomyces lunalinharesii* B-03.

For plants, the rhizosphere microbial community may be the first line of defense against soil pathogens (Zebelo et al., 2016). The interaction between antagonistic bacteria and plant rhizospheric microorganisms was traced from the point of view of antagonistic bacteria increasing root microbial diversity and revealing the biocontrol mechanism of antagonistic bacteria. The colonization of PGPR along plant roots is a prerequisite for them to execute their specific functions (Xu et al., 2019). In recent years, the research of actinomycetes was focused on its ability to control plant disease and indirectly promote plant growth. Most isolates in the genus *Streptomyces* showed surpassing antifungal activities against fungal pathogens and abilities to produce plant-growth-promoting agents in high quantity (Himaman et al., 2016). Actinomycetes could provide nutrients by the specific uptake system to stimulate plant growth (Rungin et al., 2012). Mahadevan and Crawford (1997) found that *Streptomyces olivaceoviridis*, *Streptomyces rimosus*, *Streptomyces rochei*, *Streptomyces griseoviridis*, and *Streptomyces lydicus* had the ability to improve plant growth by increasing seed germination, root elongation, and root dry weight. Uphoff et al. (2009) reported that *Streptomyces* strains significantly enhanced plant growth by increasing plant root length, number of roots, plant shoot length, number of leaves, fresh weight, and dry weight over the uninoculated control. The growth-promoting effect of *Streptomyces* is mainly evaluated from its effects on plant morphological, physiological, and biochemical indexes. Plant stem length, height, and fresh weight are intuitive indices that are easy to observe and determine and are generally used as morphological indices. The content of chlorophyll in plants directly affects the intensity of photosynthesis, and understanding the content of chlorophyll is of significance for improving plant yield.

In the present study, a pot experiment was conducted to explore the control effect of the rhizosphere growth-promoting bacteria HN6 on banana *Fusarium* wilt and the effect on soil microbial community structure. The results showed that HN6 could effectively colonize soil, release antibacterial VOCs, reduce FOC4 in the rhizosphere soil, and prevent pathogens from invading banana roots and plants (Figure 1). Among the four treatments, HN6 increased the morphological index and chlorophyll content of banana seedlings (Figure 2). In addition, HN6 showed a variety of characteristics to promote plant growth (production of IAA and siderophore and solubilization of phosphate), thus improving the nutritional status of rhizosphere soil (Figure 3). HN6 also had a certain effect on the composition of the microbial community in banana rhizosphere soil. Compared with the control, the colonization of HN6 in the rhizosphere of banana seedlings resulted in a decrease in the number of pathogenic fungi represented by FOC4 (Figure 4) and increase in the diversity of rhizosphere fungi (Figure 5). This suggested that strain HN6 decreased the relative abundance of *Fusarium* species in the rhizosphere soil to control the excessive secretion of mycotoxins that cause damage to the roots of banana seedlings. The results also showed that after infection with strain HN6, the banana seedling rhizosphere microbial community dominated by *Fusarium* changed and reorganized and evolved into a microbial community dominated by some beneficial bacteria, which is not conducive to disease occurrence (Figure 6). This may play an important role in disease prevention and control. This conclusion needs more relevant research work to be confirmed.

## Conclusion

In summary, strain HN6 was shown to produce IAA and siderophore, which can dissolve potassium and phosphate as well as fix nitrogen. After colonizing the rhizosphere soil of banana seedlings, strain HN6 inhibited the entry of pathogens into the vascular bundles and corms of banana seedlings. By reducing the relative abundance of pathogens and increasing the relative abundance of beneficial bacteria in the rhizosphere, strain HN6 could restore the rhizosphere soil microbial ecology changed by FOC4 and finally stabilize the rhizosphere soil community and control banana *Fusarium* wilt. Therefore, strain HN6 is an important microbial resource to control banana *Fusarium* wilt and promote banana growth.

## Data Availability Statement

The original contributions presented in the study are included in the article/Supplementary Material, further inquiries can be directed to the corresponding author/s.

## Author Contributions

DY, LW, and YL conceived and designed the research. DY, LW, and TW conducted the experiments. YZ and SZ analyzed the data. DY and LW wrote the manuscript. YL was responsible for funding acquisition, project administration, and resources. All authors commented, discussed, and approved the manuscript.

## Conflict of Interest

The authors declare that the research was conducted in the absence of any commercial or financial relationships that could be construed as a potential conflict of interest.

## Footnotes

1 https://www.ncbi.nlm.nih.gov/

2 http://rdp.cme.msu.edu/misc/resources.jsp
