# Supplementary material for: Plant Growth-Promoting Rhizobacteria HN6 Induced the Change and Reorganization of Fusarium Microflora in the Rhizosphere of Banana Seedlings to Construct a Healthy Banana Microflora
Source: Front Microbiol. 2021 Jul 20;12:685408. doi: 10.3389/fmicb.2021.685408 (PMC8329250; doi:10.3389/fmicb.2021.685408)
Supplement: Supplementary file 1 [file Data_Sheet_1.PDF]

Supplementary Figures and Tables

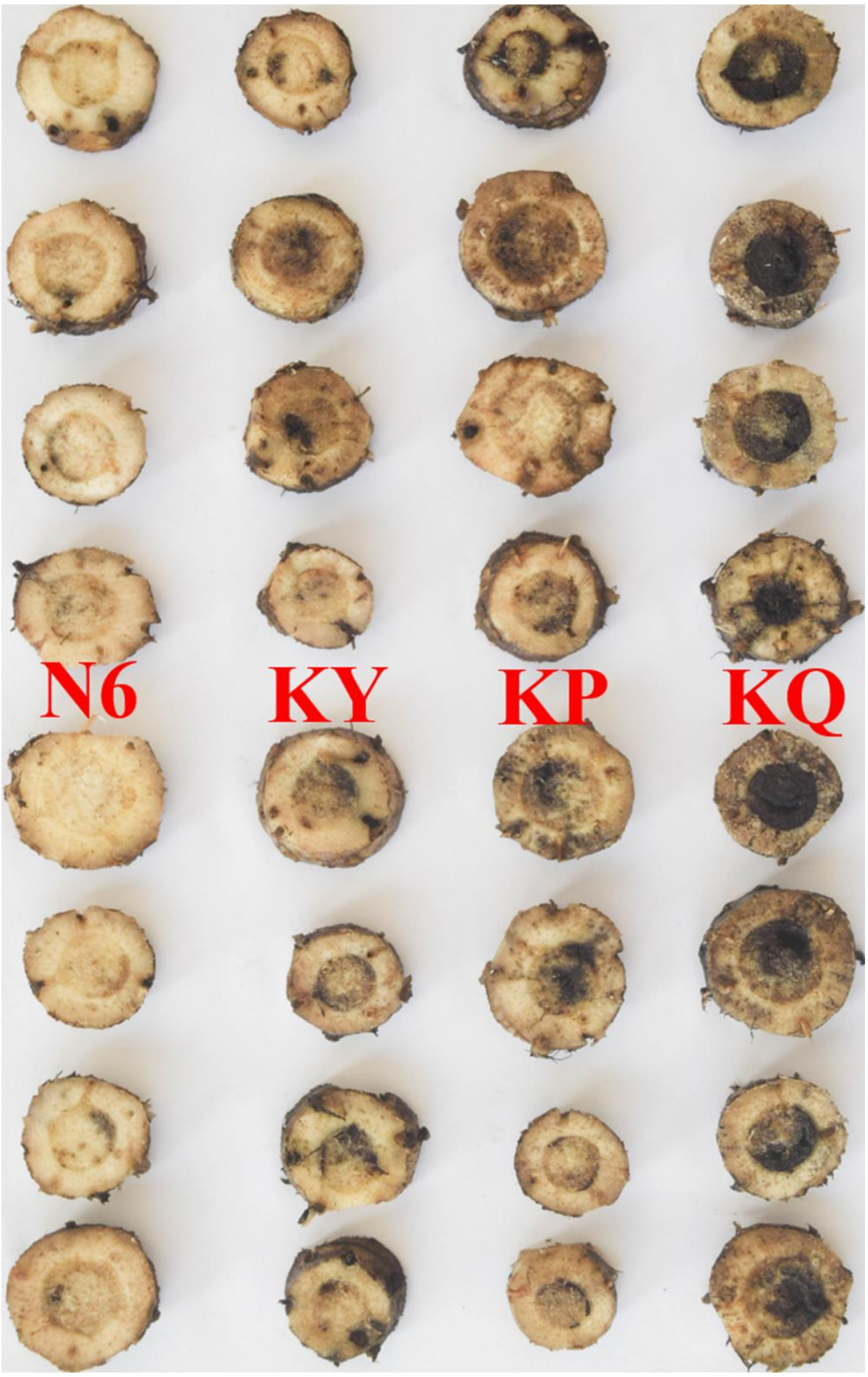

Supplementary Figure S1. The symptoms of *Fusarium wilt* in banana corms. N6 (*Foc*·TR4-GFP+HN6, 1.0×10^{7} cfu·g^{-1} soil), KY (*Foc*·TR4-GFP+0.1% carbendazim), KP (*Foc*·TR4-GFP + Gause No. 1 liquid medium), and KQ (*Foc* TR4-GFP+ sterile water).

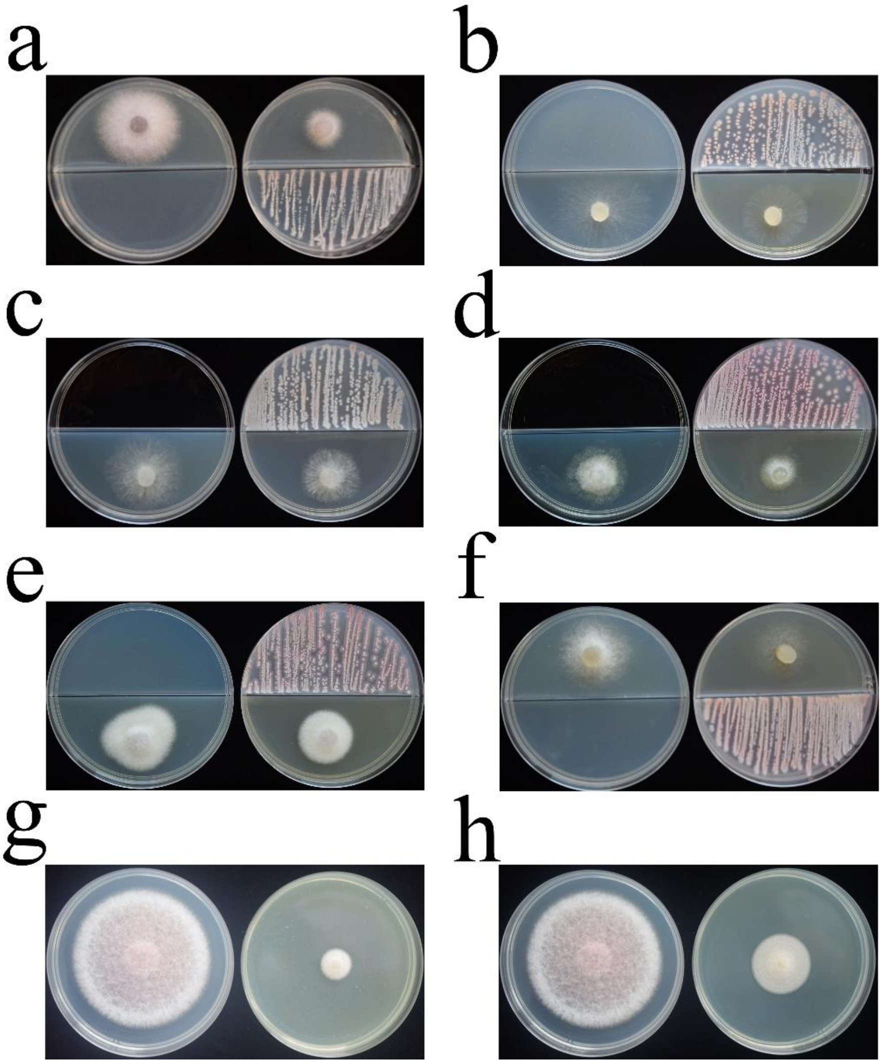

Supplementary Figure S2. Inhibitory effect of *Streptomyces* sp. HN6 volatile organic compounds on pathogenic fungi

a: *Fusarium oxysporum* f. sp.*cubense*; b:*Rhizoctonia Solani*; c:*Botryodiplodia theobromae*; d:*Colletotrichum gloeosporioides*; e:*Fusarium moniliforme*; f:*Sclerotinia sclerotiorum*; g: The VOC acridine, 9-methyl- inhibited radial hyphal growth of FOC4 at concentrations of 200 $\mu g\cdot mL^{-1}$; h: The VOC acridine, 9-methyl- inhibited radial hyphal growth of FOC4 at concentrations of 100 $\mu g\cdot mL^{-1}$

Supplementary Table 1 Separation statistics of cultivable fungi in rhizosphere soil. N6 (*Foc*·TR4-GFP+HN6, 1.0×10^7 cfu·g^{-1} soil), KY (*Foc*·TR4-GFP+0.1% carbendazim), KP (*Foc*·TR4-GFP + Gause No. 1 liquid medium), and KQ (*Foc* TR4-GFP+ sterile water). YS referred to rhizosphere soil of uninoculated plants.

Treatment	Strain number	Quantity (1.0×10^5 cfu·g^{-1} soil)
N6	N6ZJMH01 N6ZJCS02 N6ZJMH03 N6ZJMH04 N6ZJMS05 N6ZJMS06 N6ZJMH07 N6ZJMH08	8
KY	KYZJCS01 KYZJMH02 KYZJCS03 KYZJMH04 KYZJMH05 KYZJMH06 KYZJCS07 KYZJMS08	8
KP	KPZJMH01 KPZJMS02 KPZJPA03 KPZJMS04 KPZJMS05 KPZJCS06 KPZJCS07 KPZJCS08 KPZJCS09 KPZJCS10 KPZJCS11 KPZJMS12 KPZJMS13 KPZJMS14 KPZJMH15 KPZJMH16	16
KQ	KQZJCS01 KQZJMS02 KQZJPA03 KQZJCS04 KQZJCS05 KQZJMH06 KQZJMH07 KQZJMH08 KQZJMS09 KQZJMS10 KQZJMS11 KQZJMS12 KQZJMS13 KQZJMH14	14
YS	YSZJMS01 YSZJMS02 YSZJMS03 YSZJMS04 YSZJCS05 YSZJCS06 YSZJCS07 YSZJCS08 YSZJMH09 YSZJMH10 YSZJMH11 YSZJMH12 YSZJMH13 YSZJMH14 YSZJMH15 YSZJPA16 YSZJPA17	17

Supplementary Table 2 Separation statistics of cultivable bacterial in rhizosphere soil. N6 (*Foc*·TR4-GFP+HN6, 1.0×10^{7} cfu·g^{-1} soil), KY (*Foc*·TR4-GFP+0.1% carbendazim), KP (*Foc*·TR4-GFP + Gause No. 1 liquid medium), and KQ (*Foc* TR4-GFP+ sterile water). YS referred to rhizosphere soil of uninoculated plants.

Treatment	Strain number	Quantity (1.0×10^{6} cfu·g^{-1} soil)
N6	N6XJCF01 N6XJLB02 N6XJNA03 N6XJCF04<br>N6XJLB05 N6XJCF06 N6XJNA07 N6XJNA08<br>N6XJLB09 N6XJLB10 N6XJNA11 N6XJLB12<br>N6XJCF13 N6XJNA14 N6XJCF15 N6XJNA16<br>N6XJNA17 N6XJCF18 N6XJNA19 N6XJLB20<br>N6XJNA21 N6XJLB22 N6XJLB23 N6XJLB24<br>N6XJCF25 N6XJNA26 N6XJGS01 N6XJSC02<br>N6XJSC03 N6XJSC04 N6XJSC05	31
KY	KYXJNA01 KYXJCF02 KYXJCF03 KYXJCF04<br>KYXJLB05 KYXJLB06 KYXJNA07 KYXJNA08<br>KYXJLB09 KYXJCF10 KYXJNA11 KYXJNA12<br>KYXJNA13 KYXJNA14 KYXJLB15 KYXJSC01	16
KP	KPXJNA01 KPXJCF02 KPXJCF03 KPXJCF04<br>KPXJLB05 KPXJLB06 KPXJNA07 KPXJNA08<br>KPXJNA09 KPXJLB10 KPXJNA11 KPXJCF12<br>KPXJLB13 KPXJLB14 KPXJNA15 KPXJNA16<br>KPXJNA17 KPXJNA18 KPXJNA19 KPXJSC01<br>KPXJSC02 KPXJSC03	22
KQ	KQXJLB01 KQXJLB02 KQXJLB03 KQXJCF04<br>KQXJLB05 KQXJNA06 KQXJNA07 KQXJLB08<br>KQXJNA09 KQXJCF10 KQXJCF11 KQXJLB12<br>KQXJNA13 KQXJNA14 KQXJNA15 KQXJLB16	16
YS	YSXJLB01 YSXJLB02 YSXJLB03 YSXJCF04<br>YSXJCF05 YSXJLB06 YSXJLB07 YSXJLB08<br>YSXJNA09 YSXJNA10 YSXJSC01	11

Supplementary text. The strain N6FXJ01 16S ribosomal RNA, partial sequence

AGGGGGGGGTCTTACCATGCAGTCGAACGATGAAGCCCTTCGGGGTGGAT
TAGTGGCGAACGGGTGAGTAACACGTGGGCAATCTGCCCTGCACTCTGGG
ACAAGCCCTGGAAACGGGGTCTAATACCGGATATGACACGGGATCGCATGA
TCTTCGTGTGGAAAGCTCCGGCGGTGCAGGATGAGCCCGCGGCCTATCAGC
TTGTTGGTGAGGTAGTGGCTCACCAAGGCGACGACGGGTAGCCGGCCTGA
GAGGGCGACCGGCCACACTGGGACTGAGACACGGCCCAGACTCCTACGG
GAGGCAGCAGTGGGGAATATTGCACAATGGGCGAAAGCCTGATGCAGCG
ACGCCGCGTGAGGGATGACGGCCTTCGGGTTGTAAACCTCTTTCAGCAGG
GAAGAAGCGAAAGTGACGGTACCTGCAGAAGAAGCGCCGGCTAACTACGT
GCCAGCAGCCGCGGTAATACGTAGGGCGCAAGCGTTGTCCGGAATTATTGG
GCGTAAAGAGCTCGTAGGCGGCTTGTCACGTCGGTTGTGAAAGCCCGGGG
CTTAACCCCGGGTCTGCAGTCGATACGGGCAGGCTAGAGTTCGGTAGGGG
AGATCGGAATTCCTGGTGTAGCGGTGAAATGCGCAGATATCAGGAGGAACA
CCGGTGGCGAAGGCGGATCTCTGGGCCGATACTGACGCTGAGGAGCGAAA
GCGTGGGGAGCGAACAGGATTAGATACCCTGGTAGTCCACGCCGTAAACG
GTGGGCACTAGGTGTGGGCAACATTCCACGTTGTCCGTGCCGCAGCTAACG
CATTAAGTGCCCCGCCTGGGGAGTACGGCCGCAAGGCTAAAACTCAAAGG
AATTGACGGGGGCCCGCACAAGCGGCGGAGCATGTGGCTTAATTCGACGC
AACGCGAAGAACCTTACCAAGGCTTGACATACACCGGAAAGCATCAGAGA
TGGTGCCCCCCTTGTGGTCGGTGTACAGGTGGTGCATGGCTGTCGTC
